# Control Work

**Published:** 1897-09

**Authors:** 


### CONTROL WORK.

*Minnesota.* The State Board of Health, under the directorship of Dr. M. H. Reynolds, has just issued some placards especially designed to curtail the ravages of hog-cholera, glanders, and such diseases as are dangerous to the public welfare. They are to be used as a quarantine against the spread of the infection and prevent the introduction of other hogs or horses on the infected premises until after the pens and stables have been subjected to a thorough disinfection in a way satisfactory to the local boards of health, and, in the case of hog-cholera, no other hogs are allowed on the premises until six months after the last hog has died or recovered.

The authority quoted on the placards is chapter No. 233, laws of 1897: "Authority is hereby given to the State Board of Health and to the several local boards of health of the towns, villages, and cities of this State to take all steps they may severally deem necessary to control, suppress, and eradicate any and all contagious diseases among any of the domestic animals in this State, and to that end said boards are hereby severally empowered, within their respective jurisdictions, to quarantine any domestic animal which is infected with any disease or which has been exposed to infection therefrom. Any person violating any provision of this act or any rule or regulation made by the State Board of Health, or any order made by any such board under the authority hereof, shall be guilty of a misdemeanor, and be punished by a fine of not less than $25 or more than $100, or by imprisonment for not less than thirty or more than ninety days."

There has also been a special notice card issued for the safety of owners, to create a private quarantine against carelessness in neighbors or others during the prevalence of an epidemic.

The rules for controlling hog-cholera in Minnesota are as follows:

Rule 1. The following counties were more or less generally affected with hog-cholera during 1896 and 1897 up to date, and are hereby declared an infected district and designated as district "A:" Brown, Watonwan, Martin, Freeborn, Faribault, Blue Earth, Nicollet, Le Sueur, Waseca, Hennepin, and Ramsey, excepting the State Fair grounds. This district is subject to such modifications as the State Board of Health may see fit to make from time to time.

Rule 2. All that portion of the State not included in district "A" shall be known as district "B."

Rule 3. Shipment of swine from any portion of district "A" to be unloaded in district "B," and all other movements of swine, whether they be driven on foot or hauled in wagons from any point in district "A" into district "B," are hereby prohibited.

Rule 4. All shipments from points outside of this State to be unloaded within this State are prohibited, excepted as provided in Rule 5.

Rule 5. Hogs shipped from any other State into Minnesota must be crated, shipped in other than stock-cars, and accompanied by a certificate signed by a veterinarian or physician that they are free from the disease when shipped and come from an uninfected district.

Rule 6. Hogs shipped from point to point in district "B" must be crated and shipped in other than stock-cars.

Rule 7. All outbreaks of suspected hog-cholera in district "B" and in such places as may be deemed practical in district "A" shall be rigidly quarantined.

Rule 8. Shippers of hogs from point to point in district "A" shall be required by the railroad agent to sign a statement to the effect that such hogs are for slaughter only, and within five days after reaching destination. Railroad agents should preserve all certificates demanded by these rules for the protection of their companies in case any shipment of hogs should be followed by an outbreak of disease.

Rule 9. All exhibitions of swine at county fairs in district "A" are hereby forbidden.

Rule 10. All cars in which hogs are shipped into or through this State shall be constructed so as to prevent the escape of manure and litter.

These rules shall go into effect August 20, 1897, and continue in force until altered or annulled by the State Board of Health.

N. B.—These rules do not interfere with any shipments of swine for slaughter into any stockyards of the State that may be located within district "A," and these rules are so framed that the large markets of the State are located within district "A."

Statistics recently computed by the Pasteur Vaccine Company relative to the vaccination of cattle against blackleg in this country for the two years ending June 30, 1897, covering some 75,000 head of cattle, among which the previous annual mortality had averaged 10 per cent., show that since the adoption of the use of vaccine the mortality has been reduced to one-third of 1 per cent., resulting in a saving, on a conservative estimate, of $100,000. This work has covered sections of South Dakota, Wyoming, Nebraska, Colorado, Kansas, and Texas, and corroborates the results obtained in Europe prior to its introduction in this country. As this is the only remedial hope for these animals, situated as they are, remote from environments that would permit of successful treatment in times of enzoötic and epizoötic outbreaks—and even this plan of protection is accomplished only by arduous labor and difficulties—it is very gratifying to know that we are rapidly attaining effective measures for even the saving of these yearly losses.

---

The Kentucky authorities are looking after the stud licenses in that State. This is regulated by the highest fee received for service.

